# Surgical outcomes of phacoemulsification with different fluidics systems (centurion with active sentry vs. centurion gravity) in cataract patients with eye axial length above 26 mm

**DOI:** 10.3389/fmed.2025.1554832

**Published:** 2025-06-12

**Authors:** Yinan Liu, Xiaoyong Chen

**Affiliations:** Department of Ophthalmology, Peking University Third Hospital, Beijing, China

**Keywords:** high myopia, Alcon centurion, active sentry, phacoemulsification, macular vessel density

## Abstract

**Introduction:**

During cataract phacoemulsification surgery, the Alcon Centurion with Active Sentry can achieve a more stable anterior chamber, which allows a lower intraocular pressure (IOP) setting than Centurion under gravity mode. In this randomized controlled trial, we compared these two systems’ impact on high myopia patients’ macular blood perfusion under different IOP settings. To evaluate intra-op and post-op clinical performance of the Alcon Centurion with Active Sentry and under gravity mode in cataract patients with eye axial lengths > 26 mm.

**Methods:**

Fifty-two eyes of 43 cataract patients with axial lengths > 26 mm were enrolled and randomly divided into the active fluidics system (AFS) group using a Centurion Active Sentry handpiece under 30 mmHg IOP setting (26 eyes) and the gravity fluidics system (GFS) group in gravity mode under 80 cmH_2_O IOP setting (26 eyes). Intraoperative parameters, visual acuity, parafoveal macular vessel density and macular thickness were analyzed.

**Results:**

We observed no significant differences in best corrected visual acuity (BCVA), macular thickness, cumulative dissipated energy (CDE), total case time, as well as ophthalmic viscoelastic devices (OVDs) usage between the two groups. However, we found less pain complaints during surgeries and a more stable macular vessel density post-surgery in the AFS group than in the GFS group, implying higher intraoperative comfort levels and less retinal ischemia during cataract surgery using Centurion with Active Sentry under low IOP settings.

**Conclusion:**

Owing to a lower IOP setting, Centurion Vision System with Active Sentry handpiece causes less retinal ischemia and pain perception during phacoemulsification for high myopia patients.

**Clinical trial registration:**

https://www.chictr.org.cn, identifier ChiCTR2400080875.

## Background

During phacoemulsification for cataract patients, the phaco-tip releases energy and provides high perfusion in the anterior chamber, resulting in fluctuations in intraocular pressure (IOP) as well as inflammation of the intraocular microenvironment. These factors may lead to several pathological changes of the eye, including morphological changes, functional changes, and vascular remodeling (1). Previous studies have observed fluctuations in macular blood perfusion after phacoemulsification, of which by using a traditional gravity fluidics system (GFS), post-operative macular blood perfusion increases in normal eyes (2), while decreases in high myopia eyes (3). Compared with gravity fluidics system, active fluidics system (AFS) can effectively reduce anterior chamber surges and obtain lower cumulative dissipative energy (CDE) values, suggesting higher surgical efficiency and better protection for the eye (4). In the comparative study of the above two fluidics systems, it was found that the fluctuation of parafoveal blood perfusion was more obvious in GFS than in AFS (5, 6). It follows that the influence of cataract phacoemulsification surgery on macular blood perfusion should not be ignored, especially in high myopia patients who already have poor macular blood perfusion. Because compared with normal patients, phacoemulsification surgery may cause irreversible damage to the macular blood supply of high myopia patients. To this end, this study aims to evaluate the dynamic changes in the macular thickness and macular vessel density after cataract phacoemulsification with either the Centurion active fluidics with Active Sentry handpiece or Centurion in gravity mode among high myopia patients, hoping to find the best scheme to preserve their macular function.

## Methods

### Patients

This was a randomized controlled trial (RCT) and approved by the ethical committee of Peking University Third Hospital (M2023667). The study was conducted in accordance with the tenets of the declaration of Helsinki.

Inclusion criteria:

- Age related cataract patients, 40–70 years old
- AL > 26 mm
- ECD > 2,000/mm^2^
- ACD > 2.5 mm
- Lens nuclear opalescence grade 2–3 on the Lens Opacities Classification System (LOCS) III classification
- Dilated pupil > 6 mm

Exclusion criteria:

- Irregular corneal astigmatism
- Posterior staphylomas
- History of eye surgery
- Any retinal disease, macular disease that could affect VA, glaucoma, lens subluxations, eye trauma and other severe intraocular diseases

The enrolled 52 eyes (from 43 patients) were randomly divided into the AFS group and the GFS group, then accepted phacoemulsification at Peking University Third Hospital from March 2024 to November 2024. The postoperative follow-up was 1 month.

### Surgical parameters

All the cataract surgeries were performed under topical anesthesia with 0.5% proparacaine by a professional surgeon (X. Chen) using the Alcon Centurion platform with an Active Sentry handpiece for the AFS group or with an Ozil handpiece for the GFS group. The energy settings were referred to Table 1. In order to measure the intraoperative pain of the patient, a numerical rating scale (NRS) was used immediately after the surgery. Topical eye drops of 0.3% levofloxacin, 1.0% prednisolone acetate and 0.1% diclofenac sodium were used for 1 month post-operatively.

**Table 1:** Preset phacodynamic parameters.

Preset Phacodynamic Parameters	Active fluid system (*n* = 26)	Gravity fluid system (*n* = 26)
IOP	30 mmHg	80 cm H_2_O
Torsional US power^a^ (%)	60	60
Vacuum (mmHg)	500	400
AFR (cc/min)	40	40

US, ultrasonic; AFR, aspiration flow rate.

a Linear mode.

### Clinical examinations

Macular vessel density (MVD) at parafoveal region (OCTA, Optovue), macular thickness (cube volume average, CIRRUS HD-OCT, Zeiss) as well as other routine examinations were performed before surgery and at 1-day, 1-week, 1-month after surgery.

### Statistical analysis

All parameters will be summarized descriptively with mean ± SD. For normally distributed data, independent samples t-test will be used for comparison of data between groups. For non-normally distributed data, Mann–Whitney U test will be used for comparison of data between groups. Chi-square tests were performed to compare the ratios between the two groups. *p* value less than 0.05 will be considered statistically significant.

## Results

Of all the 52 cases, no complication was reported. This study observed no significant differences in baseline values between the two groups (*p* > 0.05, Table 2). And the intraoperative parameters such as the total case time, cumulative dissipated energy (CDE) and the total ophthalmic viscoelastic devices (OVDs) usage were not significantly different (*p* > 0.05, Table 3). However, the NRS pain level was greater in the GFS group than in the AFS group (*p* < 0.01, Table 3).

**Table 2:** Demographic parameters.

Demographic parameters	Active fluid system (*n* = 26)	Gravity fluid system (*n* = 26)	*p* value
Age	66.15 ± 4.92	67.43 ± 5.77	0.583
Patient number	20	23	
Gender (Male/Female)	14/ 12	13/ 13	0.781
Eye (right/left)	13/ 13	15/ 11	0.578
Stage of nucleus^a^			
2	16	18	0.560
3	10	8	
ACD (mm)	3.51 ± 0.56	3.58 ± 0.61	0.690
AL (mm)	27.30 ± 1.59	27.49 ± 1.98	0.307
CCT (μm)	554.1 ± 62.9	559.0 ± 57.8	0.612
ECD (/mm^2^)	2735.7 ± 409.5	2895.2 ± 399.4	0.495
Pre-op BCVA, LogMAR	0.35 ± 0.19	0.33 ± 0.21	0.754

a Nucleus grading by LOCSIII.

AC, anterior chamber depth; AL, axial length; CCT, central corneal thickness; ECD, endothelial cell density.

**Table 3:** Comparison of intraoperative parameters between groups.

Intraoperative parameters	Active fluid system (*n* = 26)	Gravity fluid system (*n* = 26)	*p* value
CDE (%-seconds)	5.47 ± 3.72	5.96 ± 4.92	0.523
Total case time (s)	390.4 ± 91.4	405.4 ± 104.5	0.680
OVDs usage (ml)	0.63 ± 0.49	0.78 ± 0.71	0.293
Patient pain level			
No pain	21	8	0.003
Mild pain	5	14	
Moderate pain	0	3	
Severe pain	0	1	

CDE, cumulative dissipated energy; OVDs, ophthalmic viscoelastic devices.

**Figure 1:**
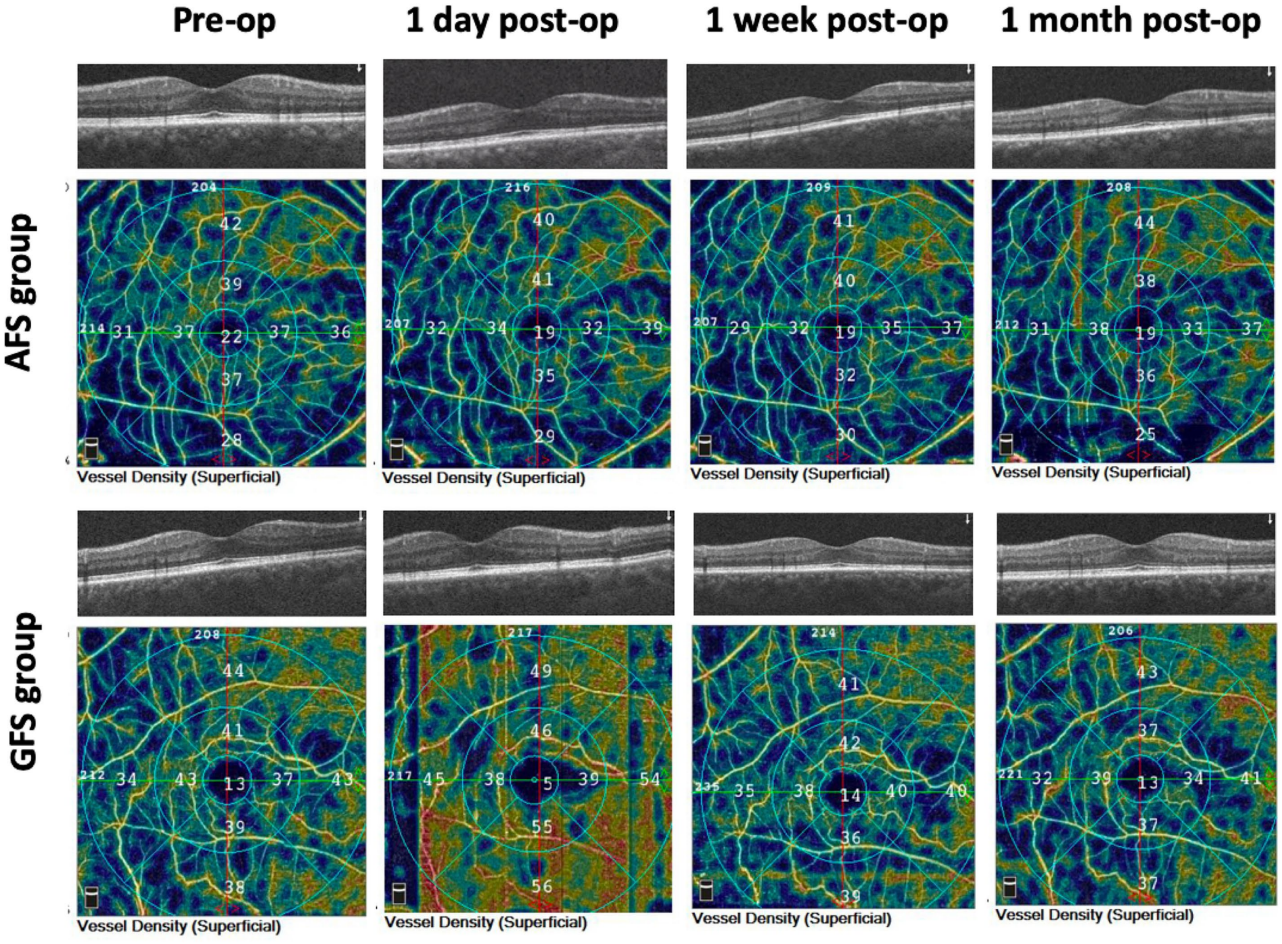
Macular OCT and OCTA results of the two groups at baseline and 3 post-operative time-points.

**Figure 2:**
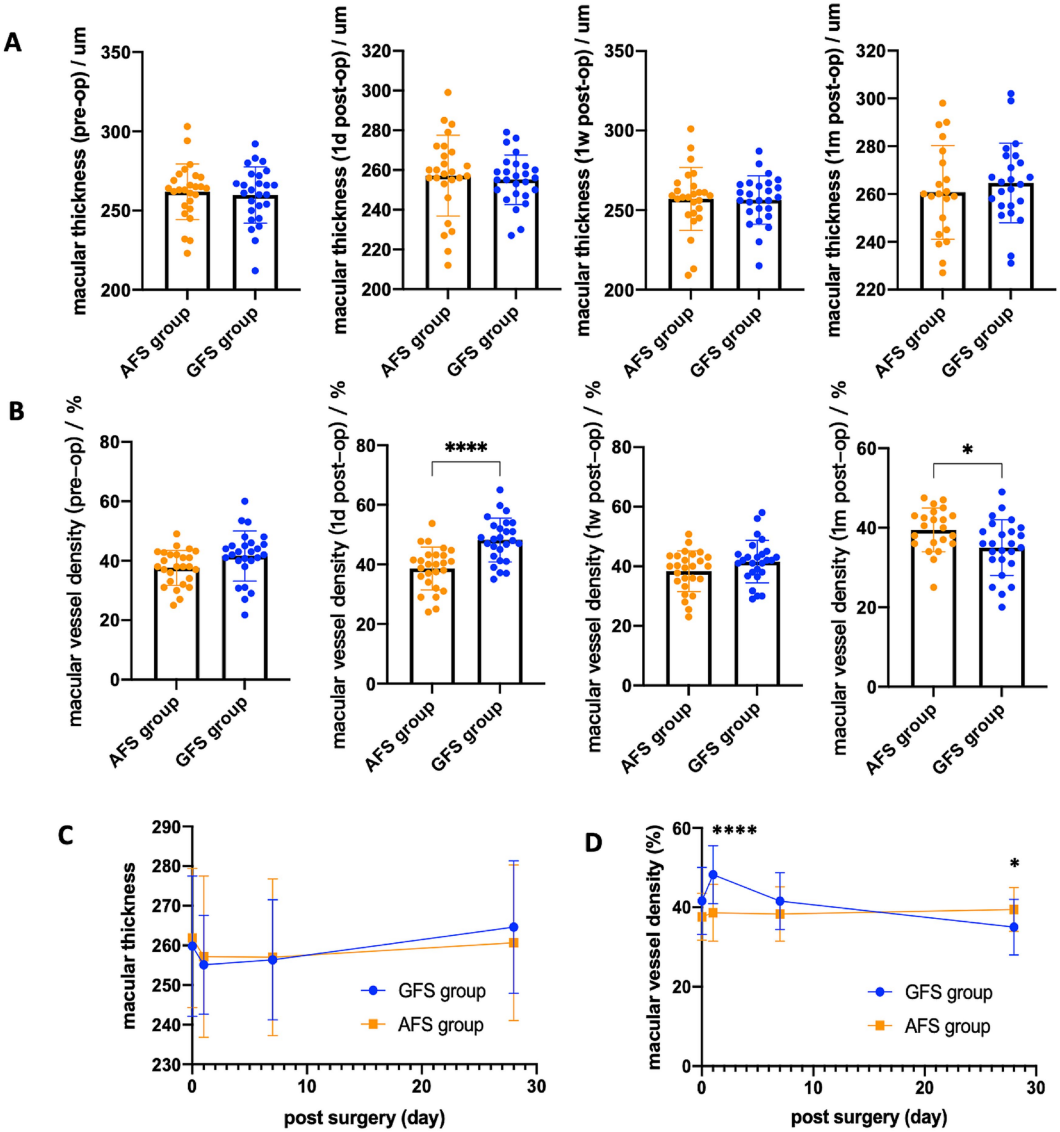
Macular thickness **(A)** and macular vessel density **(B)** of the two groups at baseline and 3 post-operative time-points. Line chart of the above statistics for macular thickness **(C)** and macular vessel density **(D)** (*p < 0.05, **p < 0.01, ** *p* < 0.001, ****p < 0.0001).

The typical examination results of the two groups were displayed in Figure 1. The parafoveal macular vessel density (MVD) in the GFS group was (48.48 ± 7.36) % at 1 day post-surgery, and significantly higher than that in the AFS group, which was (38.27 ± 7.08) % (*p* < 0.0001, Table 4; Figure 2B). The elevated MVD values in the GFS group fell back to baseline at 1 week post-surgery and then decreased to (35.08 ± 7.22) % at 1 month post-surgery, which was significantly lower than that in the AFS group (*p* < 0.05, Table 4; Figure 2B). However, compared with the GFS group, MVD in high myopia patients in the AFS group remained stable within 1 month after surgery (Figure 2D). In terms of macular thickness, both groups remained at baseline within 1 month after surgery, and no difference in macular thickness was observed between the two groups (*p* > 0.05, Table 4; Figures 2A,C).

**Table 4:** Postoperative outcomes.

Postoperative parameters	Active fluid system (*n* = 26)	Gravity fluid system (*n* = 26)	*p* value
BCVA, LogMAR			
P1D	0.10 ± 0.13	0.12 ± 0.15	0.279
P1W	0.08 ± 0.10	0.11 ± 0.10	0.315
P1M	0.04 ± 0.07	0.09 ± 0.09	0.604
CS (at 3 c/d)			
P1D	23.21 ± 15.23	21.78 ± 16.02	0.723
P1W	28.72 ± 12.95	25.44 ± 19.12	0.539
P1M	29.33 ± 13.21	26.72 ± 19.05	0.472
CCT (μm)			
Pre-op	553.2 ± 65.1	558.6 ± 56.9	0.419
P1D	559.1 ± 54.4	556.1 ± 48.0	0.527
ECD (cells/mm^2^)			
Pre-op	2732.4 ± 423.2	2931.2 ± 392.8	0.724
P1M	2552.9 ± 371.3	2601.1 ± 333.2	0.643
Vessel density at parafoveal region (OCTA)			
Pre-op	37.44 ± 5.94	41.77 ± 8.58	0.052
P1D	38.27 ± 7.08	48.48 ± 7.36	<0.0001
P1W	38.13 ± 6.90	41.20 ± 7.05	0.103
P1M	39.18 ± 5.43	35.08 ± 7.22	0.020
Macular thickness (OCT)			
Pre-op	263.08 ± 16.79	261.72 ± 15.11	0.959
P1D	258.32 ± 19.93	256.24 ± 11.30	0.659
P1W	258.04 ± 19.45	258.04 ± 19.45	0.900
P1M	259.20 ± 18.90	266.09 ± 15.41	0.468

BCVA: best corrected visual acuity, Pre-op: pre-operation, P1D: 1 day post operation, P1W: 1 week post operation, P1M: 1 month post operation, CCT: central corneal thickness, ECD: endothelial cell density.

## Discussion

**Figure 3:**
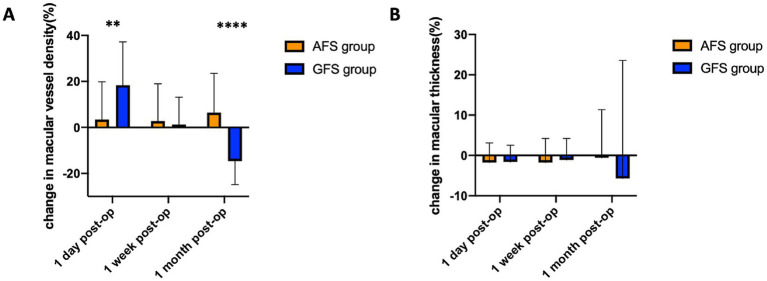
Change in macular vessel density **(A)** and macular thickness **(B)** of the two groups at 3 post-operative time-points (*p < 0.05, **p < 0.01, ***p < 0.001, ****p < 0.0001).

This study observed no significant differences in baseline values between the two groups. In terms of MVD, it remained stable within 1 month after surgery (Figure 2D) in the AFS group. However, in the GFS group, MVD increased significantly by (22.52 ± 25.27) % on the first day post-surgery, then gradually fell back, and decreased by (12.54 ± 8.91) % at 1 month post-surgery (Figures 2D, 3A). In terms of macular thickness, both groups remained at baseline within 1 month after surgery, and no difference in macular thickness was observed between the two groups (*p* > 0.05) (Figures 2C, 3B). Taken together, these results suggest that Centurion with Active Sentry provides better microvascular stability of the fundus after cataract surgery in high myopia patients than Centurion in gravity mode.

Currently, it is believed that high intraoperative perfusion and IOP will reduce retinal blood perfusion, causing transient eye ischemia and even retinal ganglion cell apoptosis (7). Findl et al. conducted the earliest study on the influence of IOP increase on human eye hemodynamics (8). This study found that in healthy eyes, a small increase of 10 to 20 mmHg IOP resulted in a significant decrease in blood perfusion of the choroid. Moreover, the decrease was more pronounced in the macular area than in the optic disc area. In addition, using OCTA to evaluate the macular blood perfusion, Li et al. (3) found that patients with high myopia who underwent cataract surgery might have a higher risk of macular ischemia based on the study results that the high myopia patients had lower superficial retinal perfusion at 1 and 3 months post-surgery compared to patients with low myopia. Similarly, Liu et al. (6) described the postoperative changes of micro-vessels around the optic papilla and macular fovea in patients undergoing cataract surgery using phacoemulsification platforms with either the AFS or GFS. And they found those vascular changes were significantly smaller in AFS group. So, the team speculated that the transient high IOP may play an important role in retinal vascular changes.

In our study, the reasons for the increase in macular blood perfusion at 1 day post-surgery and the decrease at 1 month post-surgery in the GFS group are not completely clear. However, the temporary high IOP and its fluctuation in the GFS group may play the major role. For routine phacoemulsification surgery, the most frequently used intraoperative IOP setting under traditional GFS is about 80 ~ 100cmH_2_O (approximately 59.2 ~ 74 mmHg), in order to secure the anterior chamber’s stability. In this study, we selected a relatively lower bottle height of 80cmH_2_O (approximately 59.2 mmHg) to protect the fundus. However, 80cmH_2_O (59.2 mmHg) is still much higher than the physiological IOP which is only 10 ~ 21 mmHg, and may cause irreversible damage to the eye. The rapid rise of IOP leads to a range of damage to retinal microenvironment, including retinal blood flow disturbance, ischemia–reperfusion injury, reactive oxygen species overload and increased inflammatory cytokines (7). These changes may lead to fluctuations in fundus blood perfusion and even cause vascular reconstruction. In the AFS group, the IOP setting during surgery was 30 mmHg, which was close to the physiological IOP value. And the automatic fluidics flow regulation system and Active Sentry handpiece technology of AFS could better maintain the stability of the anterior chamber and reduce the fluctuation of IOP. As a result, Centurion with Active Sentry handpiece may be more beneficial to maintain the stability of fundus vascular perfusion.

Macular thickness changes following intraocular surgeries have been shown to impair visual outcomes even in uncomplicated cases and foveal thickness changes associated with phacoemulsification have also been reported (9). Subclinical cystoid macular edema (CME) is associated with macular fovea thickening and intra-retinal cysts after cataract surgery (10). IOP fluctuation occurring in eye surgery is suspected to cause damage to the retina and phacoemulsification has also been found to increase the thickness of the retina and cystoid macular edema (11). There is a higher risk of such complication during cataract surgery due to increased fragility of zonula (12). Thus, the question is, does the perfusion pressure affect the retina? Chen et al. (13) observed in their study that compared with patients with shorter perfusion time and lower IOP setting, the macular thickness of patients with longer perfusion time and higher IOP setting increased 1 week after surgery. However, contrary to the above study, Das et al. (14) showed that the macular thickness was stable within 6 weeks after surgery concerning different intra-operative IOP settings. However, the above studies of macular edema are not centered on high myopia eyes. In our study, the effect of different IOP settings on postoperative macular thickness was not significant in high myopia patients. In other words, both Centurion with Active Sentry handpiece and in gravity mode could ensure the stability of post-operative macular thickness in patients with high myopia.

For the clinical significance of our study, as we found a significant MVD decrease of (12.54 ± 8.91) % at 1 month post-surgery in the GFS group while the MVD of the AFS group remained stable within the follow-up period, it indicates less macular ischemia after cataract surgery in the AFS group. However, because a relative short follow-up of just 1 month, the visual acuity as well the contrast sensitivity (CS) showed no significant differences among both the AFS and GFS group. To better indicates the clinical significance of this study, a longer follow-up time is requested. Based on previous studies that MVD decrease may increase risks to macular diseases such as macular atrophy, we speculate that AFS is beneficial for the macular health of high myopia patients after phacoemulsification surgery.

The main shortfall of our study is that, because of the high long-term lost rate of follow-up, our follow-up period was relatively short and was only 1 month, thus making the observation of long-term postoperative results impossible. In conclusion, owing to a lower IOP setting, Centurion Vision Platform with Active Sentry handpiece better stabilizes the macular blood perfusion and causes less retinal parafoveal ischemia after phacoemulsification for myopia patients with axial length of above 26 mm, which may be beneficial for their macular wellbeing at the long run.

## Data Availability

The raw data supporting the conclusions of this article will be made available by the authors, without undue reservation.
